# Ethical Concerns of and Risk Mitigation Strategies for Crowdsourcing Contests and Innovation Challenges: Scoping Review

**DOI:** 10.2196/jmir.8226

**Published:** 2018-03-09

**Authors:** Joseph D Tucker, Stephen W Pan, Allison Mathews, Gabriella Stein, Barry Bayus, Stuart Rennie

**Affiliations:** ^1^ University of North Carolina Project-China Guangzhou China; ^2^ Social Entrepreneurship to Spur Health Guangzhou China; ^3^ Faculty of Infectious and Tropical Diseases London School of Hygiene and Tropical Medicine London United Kingdom; ^4^ Institute of Global Health and Infectious Diseases University of North Carolina Chapel Hill, NC United States; ^5^ Department of Public Health Xi'an Jiaotong-Liverpool University Suzhou China; ^6^ Kenan-Flagler School of Business University of North Carolina Chapel Hill, NC United States; ^7^ Social Medicine Department University of North Carolina Chapel Hill, NC United States

**Keywords:** crowdsourcing, health communication, ethical analysis

## Abstract

**Background:**

Crowdsourcing contests (also called innovation challenges, innovation contests, and inducement prize contests) can be used to solicit multisectoral feedback on health programs and design public health campaigns. They consist of organizing a steering committee, soliciting contributions, engaging the community, judging contributions, recognizing a subset of contributors, and sharing with the community.

**Objective:**

This scoping review describes crowdsourcing contests by stage, examines ethical problems at each stage, and proposes potential ways of mitigating risk.

**Methods:**

Our analysis was anchored in the specific example of a crowdsourcing contest that our team organized to solicit videos promoting condom use in China. The purpose of this contest was to create compelling 1-min videos to promote condom use. We used a scoping review to examine the existing ethical literature on crowdsourcing to help identify and frame ethical concerns at each stage.

**Results:**

Crowdsourcing has a group of individuals solve a problem and then share the solution with the public. Crowdsourcing contests provide an opportunity for community engagement at each stage: organizing, soliciting, promoting, judging, recognizing, and sharing. Crowdsourcing poses several ethical concerns: organizing—potential for excluding community voices; soliciting—potential for overly narrow participation; promoting—potential for divulging confidential information; judging—potential for biased evaluation; recognizing—potential for insufficient recognition of the finalist; and sharing—potential for the solution to not be implemented or widely disseminated.

**Conclusions:**

Crowdsourcing contests can be effective and engaging public health tools but also introduce potential ethical problems. We present methods for the responsible conduct of crowdsourcing contests.

## Introduction

Crowdsourcing refers to “the practice of obtaining information or services by soliciting input from a large number of people, typically via the internet and often without offering compensation.” [1] The term encompasses a wide range of practices that were originally developed to iteratively improve commercial products based on crowd input and to change the traditional relationship between a business and a client [2]. For example, the online encyclopedia Wikipedia allows anonymous volunteers to write, edit, and manage online encyclopedia entries. Wikipedia has rapidly grown and now has 4.9 million articles that are being edited by 70,000 active contributors [3]. Crowdsourcing is used in the government and nonprofit sectors to generate innovative concepts and designs [4]. Crowdsourcing can take a wide variety of forms, including online games [5], distributed health system platforms [6], and contests to solicit new ideas [4].

Our discussion of crowdsourcing will focus on contests, also called innovation challenges, innovation contests, and inducement prize contests. Crowdsourcing contests include prize-based open contests in which individuals or teams work alone and those in which individuals work together. Contests typically include the following stages: organizing a steering committee, soliciting contributions, promoting the contest, judging contributions by experts or the crowd, recognizing excellent contributions, and sharing contributions. In the past 10 years, contests have been used to promote public health [4]. Crowdsourcing contests have been used to develop health messages [7], inform health policy [8], and improve medical diagnostics [9]. These kinds of contests can increase community engagement [7,10], improve health [10,11], and save money [11].

However, crowdsourcing contests introduce a number of potential ethical concerns [12,13], including not being sufficiently inclusive, only relying on the internet, and not disseminating the solution widely. Identifying and responding to these shortcomings is important for establishing crowdsourcing as a force for the public good and as a useful public health tool. These concerns have received limited attention in the public health literature on crowdsourcing to date [2,4]. This paper describes crowdsourcing contests by stage, describes common ethical challenges, and provides guidance on implementing crowdsourcing contests ethically.

## Methods

We conducted a scoping review [14] to synthesize literature on the ethical conduct of health-related crowdsourcing projects. This review includes applied and theoretical ethics literature related to crowdsourcing research and practice. Scoping reviews allow one to examine the literature in a structured way but are different from systematic reviews in their methodology and content [15]. Our review focused on sources between January 1, 2005 and July 1, 2017. We examined a wide range of anthropological, ethical, social science, and related literature on crowdsourcing to promote public health. We anchored this discussion in a particular example of a single crowdsourcing contest. In addition, we examined several crowdsourcing contest failures to understand concerns and potential ethical problems.

We identified studies using keyword searches in electronic databases, including MEDLINE (OVID interface, 1946 onwards), Google Scholar, expert opinion, and Wikipedia. For database searches, we used phrases and synonymous variations of the following terms: crowdsourcing, innovation challenge, ethics, implementation ethics, and applied ethical analysis. We also identified studies based on searches of reference lists, hand-searching key journals identified from initial database inquiries, and unpublished conference abstracts. We prioritized studies that examined crowdsourcing contests in health contexts. Our search included studies that provided empirical or theoretical data on crowdsourcing contests in the past 12 years.

## Results

### Overview

Our scoping review data are organized according to the 6 stages of a crowdsourcing contest—organizing, soliciting, promoting, judging, recognizing, and sharing [7,10]. First, the contest organizers form a contest steering committee to articulate the purpose, values, and methods of the contest. Second, an open call for content (eg, concepts, images, videos, or other materials) is announced via in-person events and social media. This open call clarifies the goals and terms of the contest, the prize or incentive structure, and the nature of participation. The open call plays a key role in defining the crowd. Third, the crowd is iteratively engaged through feedback sessions, in-person events, and social media. Fourth, a group of judges evaluates each contribution based on prespecified criteria to determine finalists. In some cases, the judges are the crowd itself. Finalists and others are awarded prizes according to their rank order. The judging process aggregates crowd wisdom [16]. Fifth, contest finalists are announced and recognized through an incentive structure. Sixth, the steering committee shares the finalist solution(s) with the community. After discussing each of these 6 stages, we review the literature on failures in crowdsourcing and discuss ethical principles of crowdsourcing contests.

### Organizing a Steering Committee

The first step of a crowdsourcing contest is to establish a steering committee that will decide the structure and function of the contest. The steering committee powerfully shapes the contest and provides a set of norms, expectations, and deadlines. Often contests are divided into ones that focus on engaging large numbers of the community or on resulting in a high-quality outcome [4]. The condom video contest in China was focused on creating a high-quality video. The condom video contest steering committee was composed of youth, community health leaders, men who have sex with men, doctors, business leaders, and researchers. The group was organized by Sesh Global, an organization with experience in crowdsourcing contests. The steering committee met on a monthly basis to discuss the scope, rules, and promotion of the contest. In addition, the steering committee used email and social media to discuss contest developments.

One potential ethical problem with organizing a steering committee is the possibility of excluding community members or voices that are important to the contest. Often individuals from marginalized, vulnerable groups who lack a voice in decision making are less likely to be represented on steering committees. This problem has important implications for contests because the steering committee establishes the expectations and rules governing the entire process. For example, a contest focused on gay men and HIV ought to include gay men and people living with HIV. A committee lacking appropriate representation of key groups could undermine both effectiveness and trust in the contest.

One way to mitigate the risk of excluding important community voices is to have transparent criteria for selecting steering committee members. In addition, aligning the composition of the steering committee with the overall purpose of the contest could help ensure that community voices are represented. Given local power dynamics related to nonexpert advice, it may also be useful to have local, in-person meetings of the steering committee specifically to establish trust and align expectations.

### Soliciting Contributions

Contest organizers design the open call soliciting contributions. The call for contributions is open so that anyone can contribute. Open calls can be through social media, in-person, or both. We define social media as websites or apps that allow users to create and share content or to engage in social networking [17]. Our condom video contest call for entries in China shows how the language, format, and structure shape a crowd (Multimedia Appendix 1). The call for entries was distributed through social media and in-person events at local high schools, colleges, and community-based organizations. The choice of distribution channels encouraged young Chinese individuals to participate but allowed entries from anyone.

One potential problem with calls for entries is over-reliance on social media announcements and insufficient attention to in-person events. Most private sector contests have focused on using social media calls to solicit entries [18], including several calls exclusively through social media [19,20]. There are two ethical problems with exclusively social media open calls. First, there is still a substantial digital divide between those who use social media and those who do not. Individuals who use social media tend to be from more developed regions or sectors and have higher socioeconomic status compared with those who do not use social media [21]. An exclusively social media call would not only constrain participation among some vulnerable groups, but it could worsen some of the entrenched social inequities. Second, among those who use social media, there is a further barrier to engaging sophisticated contest platforms, such as Ideascale, a company that creates online platforms for crowdsourcing contests [22]. A wide range of these platforms have been developed to crowdsource tasks. However, individuals who have the skills, knowledge, and experience to participate in these online platforms are a subset of the crowd, skewing its composition and unfairly excluding those without these skills but who are interested and could meaningfully contribute.

Careful attention to soliciting contributions can help to deal with these problems. In-person contest promotion events are one mechanism to broaden access to contests and diversify the crowd. These events have been used in several health contests [7] and have been found to increase participation and quality of participant entries. In-person events could take the form of classroom didactics, interactive feedback sessions, or community-led events. Capacity building sessions [23] could help individuals to learn about contributing on social media platforms. In addition to in-person contest promotion, ensuring multiple channels for contributing would be useful. This could include providing contributions through mail, in-person, or short text message. Contests should be as inclusive as possible relative to the intended audience. It is important to note that the goal is not for universal participation but to provide an opportunity to participate to those who would have a reasonable expectation of contributing to the contest.

### Promoting Crowd Engagement and Contributions

Following the open call, there is an iterative process of engagement between organizers and potential participants. Our condom contest organized in-person and social media engagement activities to promote submissions. This included integration of in-person and social media activities so that they complemented each other. Approximately three-quarters of those who submitted to the contest participated in at least one engagement activity. These activities established trust in the contest, built confidence in contributing, and established social norms about how to participate in the contest. Engagement activities avoided giving examples in order to decrease cognitive fixation and increase innovation [24,25]. However, this stage of crowdsourcing contests also raises potential ethical concerns. Disclosure of confidential information by contributors and the possibility of social media trolling are two primary concerns.

Authentic engagement in a contest allows those who contribute to draw on their own unique talents, preferences, and local social context. However, this personal process introduces the risk of private information being divulged, often unintentionally, as part of engagement and contributing. These concerns have been raised more generally in the crowdsourcing literature [26]. In our case, some condom videos included identifiable individuals. The contest organizers had clear guidelines establishing that all videos could be publically viewed and any individual who participated in the video gave permission to be included. This decreased the risk of unintended disclosure associated with viewing the videos. In addition, contest organizers may consider having more stringent requirements about obtaining written consent for an individual’s photograph or other personal information to be included in the contribution or going back to finalists to confirm consent before video or other forms of dissemination.

In addition, social media trolling has been reported within crowdsourcing contests. The word troll comes from the Scandinavian mythology, referring to evil small creatures who disturb travelers [17]. Today the term “trolling” refers to individuals who (usually anonymously) harass, provoke, or insult others online [27]. Trolling has been reported in a range of social media contexts [28,29], including contests [30,31]. Although trolling may be largely protected in some countries by the right to free speech [32], organizers of crowdsourcing contests should make efforts to anticipate these harms and provide protection. This type of ethical concern can to some extent be addressed through online platform moderation and algorithms for detecting offensive words, as well as informing participants of potential risks of trolling during the consent process.

### Aggregating Crowd Wisdom: Judging Contributions

Once the crowd has engaged in the contest and submitted contributions, these contributions are judged. The condom contest videos were evaluated by a multisectoral group of local people living with HIV, youth, physicians, and public health experts. Local judging increased community ownership of the contest and increased the likelihood that local characteristics (eg, using the local dialect) would be incorporated. At the same time, the nature of judging brings up a range of potential ethical issues, including how to fairly select judges. One common approach to judging contests has been to involve the crowd in judging entries, cited as a cost-effective way to engage potential participants [33]. However, if the crowd is exclusively defined online, it would be prone to the same problems described above in addition to bias, inconsistent judging criteria, and favoring popular opinion [34]. Crowd evaluation may also lead to voting based on criteria not consistent with the goals of the contest. For example, the British contest to name a government research vessel resulted in the entry “Boaty McBoatface” receiving 124,109 votes, more than fourfold greater than the next entry [35]. Organizers found themselves in the dilemma of accepting an absurd name or rejecting a crowdsourced outcome. They eventually compromised by using “Boaty McBoatface” to name a submersible carried by the research vessel dubbed the *Sir David Attenborough* [36]. In addition, the contest contributor with the largest number of online followers may be more likely to receive votes in support of their contest entry. Empirical evidence from private sector contests confirms that online crowd evaluation is biased toward individuals with greater social networks compared with expert judge evaluation [33]. Two studies found that individuals who win crowd-judged prizes are not as likely to sustain their engagement over time compared with individuals who win expert-judged prizes [33,37].

When a crowdsourcing contest has a relatively low number of entries (<100), a panel of expert judges could evaluate contributions. Judges would need to be selected in a fair way that is consistent with the mission and goals of the overall contest. Elements from “fair process” procedures—which emphasize transparency, justification of rationales, opportunities to appeal decisions, and so on—could help in the constitution of the judging panel and also help guide the decisions they make [38]. This could include evaluation of the judge panel to help ensure that a broad range of judges are represented and decrease reliance on social media. Several private sector contests demonstrate the feasibility of having a judging panel evaluate contributions [39].

### Celebrating Crowd Wisdom: Recognizing Contributions

Following the judging process, contributions can be recognized by prizes or incentives, acknowledgment, and retention of legal rights to products created. Incentive structures for crowdsourcing vary based on the goals and missions of the contest. Some contests have a single large prize [40] while others recognize a number of contributions [41]. The condom contest included individual prizes for the top three contributors as well as participation prizes. The top contributors were announced on social media as a further form of recognition. All contributors retained the rights to their videos (those who submitted the videos could use them for any purposes), consistent with the goal of the contest to promote community agency.

An ethical challenge related to recognition in crowdsourcing contests is the potential for exploiting those who make substantial contributions. Insufficient recognition of those who contribute to contests has been noted in many online contest settings [42-44]. The condom contest decreased the likelihood of this exploitation because there were several formal and informal ways of recognizing participants, alongside retention of their legal rights. The contest also shared the finalist video online in several forums.

Appropriately recognizing contributions provides a way of addressing these concerns about crowd exploitation related to the incentive structure and acknowledgment. First, clearly stating during the consent process how contributions will be recognized can mitigate exploitation to some extent. Second, incentive structures with multiple prizes (of different types) promote a broad spectrum of participation. Including special prize categories that focus on participation rather than merit have been used in some contests [7]. Third, formally acknowledging and celebrating contributions are important. Several studies have shown that intrinsic benefits of participation (such as recognition and media attention) are more important than extrinsic benefits in the context of crowdsourcing [10]. Governance of ownership and permissible uses of finalist contributions may also minimize exploitation.

### Sharing and Implementing the Solution With the Community

The final stage of a crowdsourcing contest is to share the solution more widely with the community that contributed. Henk van Ess has argued that crowdsourcing must give back to the public and share the solution more widely [45]. In this way, crowdsourcing reciprocates in a commensurate way to what the community contributed. Other crowdsourced research has suggested that perceptions of fairness are important for those contributing to crowdsourcing projects [46,47]. Our condom video contest provided prizes to finalists, participation prizes, and then made the videos available on public platforms in China.

Limited sharing of the crowdsourced solution presents an important ethical concern associated with crowdsourcing contests. This also differentiates public-oriented contests from their private sector counterparts. Most private sector contests see the finalist solutions as their own intellectual property; the terms of many private contests give intellectual property rights to organizers. Limited sharing could take the form of only describing contests in articles that are inaccessible to nonsubscribers behind a paywall.

Clearly establishing a plan early in the process for sharing and prizes as part of the call for entries can mitigate the risk of insufficient sharing. While having clear sharing expectations is important, there should also be sufficient flexibility to give the steering committee ultimate authority in making final decisions. For example, the condom contest mandated sharing of the final video on regional, national, and international networks. The call for entries specified this plan, in addition to a plan to recognize excellent entries. The benchmark for “excellent” was decided by the steering committee.

### Learning From Failures

Previous examples of crowdsourcing contests that were partially or incompletely effective provide guidance (Table 1). In 2013, the condom manufacturing company Durex invited the public to vote on which city in the world should receive a special condom rush delivery service [48]. By the end of the contest, the nonexistent city of “Batman” in Turkey had received the most votes, and contest organizers were faced with the ethical dilemma of blatantly rejecting the clear will of the crowd or endorsing a deliberately facetious winning entry. Such instances of crowd hijacking are not uncommon [49], and contest organizers should prepare for scenarios where the crowd may use the contest platform to advance an agenda that deviates from that of the contest organizers’. Given the unpredictability of the crowd, it is important for organizers to clearly explain their rights to prospective participants, including the right to deem certain kinds of entries inadmissible.

Past contests have also shown that successfully developing products through crowdsourcing is not formulaic, and that breakthrough innovations are by no means guaranteed. The start-up company Quirky had managed to secure hundreds of millions of investment dollars to develop innovative household consumer products through open online contests [50]. However, despite deep financial resources and hundreds of thousands of contest contributors, Quirky failed to produce any radically innovative products and eventually declared bankruptcy 6 years after its founding. One of the major problems was that Quirky innovators had disagreements with the company in the late stages of business development [50]. This miscommunication could be avoided by involving community members earlier in the process of development.

Finally, a German contest solicited public input on a ban on circumcision. A political party decided to crowdsource local opinions on the topic, targeting the area of North Rhine-Westphalia. Despite having a population of 18 million individuals, the contest only received 20 submissions [51]. This underscores the importance of having a steering committee that plans in advance and understands community interests and willingness to take part in crowdsourcing contests.

### Ethical Principles in Crowdsourcing Contests

We identified several general reviews that broadly considered ethical principles associated with crowdsourcing contests [12,13,52]. These highlighted theoretical concerns about privacy, accuracy of information, property, and accessibility in the context of computer science. However, this limited literature did not focus on health contests.

**Table 1:** Implementation ethics issues and potential solutions associated with crowdsourcing contests.

Contest stages	Implementation ethics issue	Potential solution
1 Organizing	Lack of input from community voices or marginalized groups	Explicitly state criteria for selecting steering committee members to ensure adequate representation
2 Soliciting	Online contests limit participation to a subset of internet-using individuals	In-person events to promote contests; multiple ways of receiving contributions
	Social networking sites narrow participation in contests to a subset of social media-savvy individuals	Allow contributions via email, in-person, cell phones, and other forms that do not require online access or social media
3 Promoting	Public contributions may include confidential or private information	Clear contest guidelines that clarify whose permission has been obtained and potentially enhanced consent process before dissemination
	Social media platforms for contributing may introduce opportunities for online harassment	Social media moderators and algorithms for detection of explicit language
4 Judging	Crowd evaluation may be biased in favor of online individuals with larger social networks	Form a local judge panel composed of key individuals representing different perspectives or backgrounds
	Multiple ways of selecting judges	Establish guidelines for selecting judges and transparent procedures for evaluation and judging
5 Recognizing	Single prize contests that are most optimal provide no recognition for most contributors	Multiple prize or incentive structure encourages a broad range of participation
	Online contests may not sufficiently recognize contributions	In-person prize announcements
6 Sharing	More is taken from the community than given back	Establish a formal mechanism to share or implement the solution more widely with the local community

## Discussion

### Principal Findings

Our review suggests that crowdsourcing contests for public health introduce several potential ethical concerns. These concerns can be categorized within the 6 stages of crowdsourcing contests—organizing, soliciting, promoting, judging, recognizing, and sharing. Our analysis suggests that these concerns can be minimized with appropriate planning and consultation. Our review expands the limited literature on crowdsourcing contests for public health [4] by focusing on ethics, examining an empirical case, and including a formal scoping review.

Our data suggest that several ethical concerns associated with crowdsourcing contests can be anticipated and avoided. For example, the awkward situation of having crowds decide on a trivial name such as *Boaty McBoatface* can be avoided by separating the process of soliciting names and choosing names eligible to be voted on. Other risks can be mitigated through appropriate contest planning. For example, inviting a diversity of local steering group members can increase the likelihood of local community perspective representation. Other ethical concerns are related to the use of social media within contests (eg, privacy concerns) and have been well described elsewhere [53]. All of these types of ethical concerns underscore the need for organizers of crowdsourcing contests to include sufficient time for planning and designing a contest.

Our scoping review did not identify studies that articulated ethical principles of crowdsourcing contests for health. This may be because few studies have focused on using crowdsourcing contests to improve public health [4]. This also may be related to the breadth of diversity of crowdsourcing contests activities, including research studies, community engagement programs, and communications strategies. However, our analysis suggests that there are several shared contest stages each of which has potential ethical concerns.

Crowdsourcing contests have implications for public health research and policy. In terms of research, further empirical study on ethical problems associated with crowdsourcing contests is necessary. Such research could help to refine the method and increase the likelihood of crowdsourcing contests achieving their goals. This research could include qualitative studies of those participating and organizing crowdsourcing contests [54]. Such research would provide valuable input for a future ethical framework specific to crowdsourcing. In terms of policy, crowdsourcing contests could help to inform public health policy. The multisectoral, transparent, and open nature of contests establishes a strong foundation for policy making. For example, the World Health Organization (WHO) used a crowdsourcing contest [55] to solicit descriptions of hepatitis testing that were directly included in the 2017 WHO hepatitis testing guidelines [56]. Given the potential for contests to inform policy, more formal principles and ethical considerations associated with crowdsourcing contests may be useful.

### Limitations

Our analysis has several limitations. First, most health contests have been single events and have yet to be serialized and formally incorporated into routine public health practice. Serial contests are likely to have different ethical challenges and may be substantially different in terms of implementation. Second, health-focused crowdsourcing contests are relatively new and while there are many examples of health-related contests, few are formally evaluated using validated metrics. Our analysis focused on a single example. Further implementation research is needed to define the most efficient and responsible use of crowdsourcing contests. Third, there is variation in the extent to which contests are driven by crowd input. Some health projects involve the community at all stages [57], while others have more intensive community input only at the start of the project [4].

### Conclusions

Crowdsourcing contests may be a useful tool to develop inclusive public health programs but also pose ethical concerns at each stage. Unraveling these ethical concerns requires careful planning, consideration, and consultation. Our analysis provides several practical steps for the responsible conduct of crowdsourcing contests and identifies areas for future research.

## Appendix

Multimedia Appendix 1 Call for entries from the crowdsourcing contest.

## Abbreviations

IRB: Institutional Review Board
WHO: World Health Organization
