# Supplementary material for: Ethical Concerns of and Risk Mitigation Strategies for Crowdsourcing Contests and Innovation Challenges: Scoping Review
Source: J Med Internet Res. 2018 Mar 9;20(3):e75. doi: 10.2196/jmir.8226 (PMC5866301; doi:10.2196/jmir.8226)
Supplement: Multimedia Appendix 1 [file jmir_v20i3e75_app1.pdf]

Additional File 1. Call for entries from a condom promotion video contest in China, 2015 (translated from Mandarin Chinese).

SESH is calling for creative one-minute videos promoting condom usage! Condoms can be bought anywhere, from “7-11” stores to vending machines, but there are still embarrassment and taboos about buying them and most importantly, using them. We are calling for a video that can change how people view using condoms. Finalist videos will be promoted on local, regional, national networks.

We are accepting videos from May 1- June 7th! Please submit your video through our website by filling out the submission form: xxxxxx

Purpose:
1) To increase condom use among those who do not use condoms

Judging Criteria:
1) capacity to increase condom use
2) capacity to be share-able or “go viral”
3) provide value to the individual.

In-person Events

May 7th
Guangdong Pharmacy University, University city- 11:30am-6pm
Sun Yatsen University, Medical Campus- 7pm-9pm

May 18
Guangdong Traditional Medicine University 11:30-5:30pm

May 19
South China Normal University- 7:30pm-9:00pm

June 1
Southern Medical University

Workshops

May 16th
Mega University City, Guangzhou

If you are interested in holding an event, please email xxxxxx !

Follow our WeChat and Weibo for more information!
WeChat： Seshchina
Weibo： weibo.com/seshglobal

Website： www.seshglobal.org
